# Practice and predictors of self-care behaviors among ambulatory patients with hypertension in Ethiopia

**DOI:** 10.1371/journal.pone.0218947

**Published:** 2019-06-26

**Authors:** Yirga Legesse Niriayo, Seid Ibrahim, Tesfaye Dessale Kassa, Solomon Weldegebreal Asgedom, Tesfay Mahari Atey, Kidu Gidey, Gebre Teklemariam Demoz, Desalegn Kahsay

**Affiliations:** 1 Department of Clinical Pharmacy, School of Pharmacy, College of Health Sciences, Mekelle University, Mekelle, Tigray, Ethiopia; 2 Clinical Pharmacy and Pharmacy Practice Unit, Department of Pharmacy, College of Health Sciences, Aksum University, Aksum, Tigray, Ethiopia; Fordham University, UNITED STATES

## Abstract

**Background:**

Despite the benefits of evidence-based self-care behaviors in the management of hypertension, hypertensive patients have low rate of adherence to the recommended self-care behaviors. Studies related to self-care behaviors among hypertensive patients are limited in Ethiopia.

**Objective:**

To assess the rate of adherence to self-care behaviors and associated factors among hypertensive patients.

**Method:**

A cross-sectional study was conducted at the cardiac clinic of Ayder comprehensive specialized hospital among ambulatory hypertensive patients. Self-care behaviors were assessed using an adopted Hypertension Self-Care Activity Level Effects (H–SCALE). Data were collected through patient interview and review of medical records. Binary logistic regression analysis was performed to identify predictors of self-care behaviors.

**Result:**

A total of 276 patients were included in the study. The majority of the participants were nonsmokers (89.9%) and alcohol abstainers (68.8%). Less than half of the participants were adherent to the prescribed antihypertensive medications (48.2%) and recommended physical activity level (44.9%). Moreover, only 21.45% and 29% were adherent to weight management and low salt diet recommendations, respectively. Our finding indicated that rural resident (adjusted odds ratio [AOR]: 0.45, 95% confidence interval [CI]: 0.21–0.97), comorbidity (AOR: 0.16, 95% CI: 0.08–0.31), and negative medication belief (AOR: 0.25, 95% CI: 0.14–0.46) were significantly associated with medication adherence. Female sex (AOR: 0.46, 95% CI: 0.23–0.92), old age (AOR: 0.19, 95% CI: 0.06–0.60) and lack of knowledge on self-care behaviors (AOR: 0.13, 95% CI: 0.03–0.57) were significantly associated with adherence to weight management. Female sex (AOR: 1.97, 95% CI: 1.03–3.75) and lack of knowledge on self-care (AOR: 0.07, 95% CI: 0.03–0.16) were significantly associated with adherence to alcohol abstinence. Female sex (AOR: 6.33, 95% CI: 1.80–22.31) and khat chewing (AOR: 0.08, 95% CI: 0.03–0.24) were significantly associated with non-smoking behavior. There was also a significant association between female sex and physical activity (AOR: 0.22, 95% CI: 0.12–0.40).

**Conclusion:**

The rate of adherence to self-care behaviors particularly weight management, low salt intake, physical exercise, and medication intake was low in our study. Elders, females, khat chewers, rural residents, and patients with negative medication belief, comorbidity, and inadequate knowledge of SCBs were less adherent to self-care behaviors compared to their counterparts. Therefore, health care providers should pay more emphasis to patients at risk of having low self-care behaviors.

## Introduction

Hypertension (HTN) is a chronic medical condition that can be defined as a persistent elevation of systolic blood pressure (BP) ≥ 140 mm Hg and/or a diastolic BP ≥ 90 mm Hg [1]. HTN is a global public health problem that affects more than a quarter of the global adult population [2]. Globally, the prevalence of HTN continues to increase and its prevalence is projected to be 1.56 billion by 2025 [3–5]. The prevalence of HTN has shifted from developed countries to developing countries since the past four decades [6, 7]. Almost three-fourths of the people with HTN live in developing countries [8]. Currently, the African region has the highest prevalence of HTN with an estimated prevalence of 46% [9, 10]. Likewise, the magnitude of HTN has been increasing in Ethiopia. According to the finding reported in 2014, HTN was the most prevalent non-communicable disease with an overall prevalence of 19.6% in Ethiopia [11].

Uncontrolled HTN is a major risk factor for cardiovascular diseases (CVDs) and renal diseases [12]. HTN is responsible for about one-half of all deaths from stroke and heart disease [3, 13]. Complications of HTN have been estimated to cause about 9.4 million deaths every year, which accounts for 17% of total deaths worldwide [3, 14, 15]. Furthermore, it contributes for the losses of 143 million disability-associated life-years [3, 14].

Self-care behavior (SCB) is an important activity undertaken by an individual in order to improve health or prevent disease [16]. The critical components of hypertension SCBs include adherence to medications and lifestyle modifications involving diet, exercise, smoking cessation, and alcohol abstinence [17, 18]. SCBs are crucial for the prevention and management of HTN [1, 17]. Collective evidence revealed that adherence to SCBs lowers BP, increases the efficacy of antihypertensive medications, and reduces the complications and overall mortality associated with HTN [17, 18].

Despite the availability of several effective pharmacologic and non-pharmacologic therapies, HTN control remains suboptimal worldwide [18–21]. Many factors contribute to the poor control of HTN [22, 23], of which, non-adherence to HTN SCBs is the leading cause of poorly controlled HTN [23–26]. Patients’ adherence to SCBs is crucial to control HTN. However, several studies reported a low rate of adherence to the recommended SCBs [27–32].

In Africa, there is a huge gap in the treatment and control of hypertension [15, 33]. Although HTN is modifiable and treatable, awareness about HTN management is very low in developing countries [34, 35]. Many of the hypertensive patients in Africa were ignorant of their disease and treatment [33, 34] owing to the lack of access to healthcare, mistrust in western medicine, and inadequate health illiteracy [36–38]. Despite adherence to SCBs is a crucial part of patient care to achieve the desired goal of therapy, the practice of self-care activities remained poor in African population [39].

Many factors including socioeconomic status, belief about medications, comorbidity, availability of medications, access to healthcare, level of health literacy, number of medications, duration of therapy, age, gender, culture, educational status, and knowledge of the disease and treatment have been associated with the rate of adherence to SCBs [19, 31, 35, 40–42]. Different studies reported that the use of alternative healing agents, belief of HTN curability by traditional and faith healers, and low level of health literacy were significantly associated with non-compliant health related behaviors in African population [43, 44]. Health surveys in Africa reported that about two-thirds of the African population had a low level of health literacy [43]. In Africa, people still believe that HTN is caused by a spiritual power that cannot be effectively treated with western medicine [44, 45]. In developing countries including Ethiopia, belief in western medicine is poor because a significant proportion of the patients still depend on traditional and faith healers [44, 46, 47]. Therefore, the negative attitude toward western medicine significantly affects the compliance to HTN treatment [44, 48, 49].

Globally, most of the current studies focus mainly on anti-hypertensive medication adherence [50]. However, adherence to the other important components of SCBs including low salt diet, exercise, weight management, smoking cessation, and alcohol abstinence have been neglected [50]. Likewise, despite the proven benefit of SCBs in BP control, little is known regarding the practice of SCBs and associated factors in Ethiopia. Hence, it is very crucial to conduct such kind of studies in countries like Ethiopia where there are limited healthcare resources, poor health care system, and low level of health literacy in order to develop strategies to improve SCBs and achieve better control of HTN. Therefore, the present study aimed to investigate the practice of SCBs and associated factors among hypertensive patients in Ethiopia.

## Materials and methods

### Participants

The source population for this study was all patients with hypertension who had follow-up at the cardiac clinic of Ayder comprehensive specialized hospital (ACSH). All patients with HTN who visited the hospital between January 2017 and March 2017 and fulfilled the inclusion criteria were included in the study. The criteria for inclusion were a diagnosis of hypertension, age >18 years, and duration of follow-up ≥6 months with at least one antihypertensive medication. The exclusion criteria were unwillingness to give consent, serious illness to complete the interview, and incomplete patient’s medical records.

The sample size was calculated using a single population proportion sample size estimating formula. For population ≥10, 000, the formula can be given as; n = [(Z_1-α/2_)^2^ p (1-p)]/d^2^, where, n = minimum sample size, Z_1-α/2_ at 95% confidence level = 1.96, P = estimated prevalence of SCBs among patients with HTN (50%), d = Margin of error to be tolerated (0.05). Substituting all in the above formula, n = 384. Since the total population in our study was <10,000 (1004), the sample can be recalculated using correction formula as follows; Nf = n/(1+n/N), where, n = minimum sample size (384), Nf = actual sample size using correction formula, N = actual population size (1004). Therefore, substituting all in the above formula, Nf = 278. Taking 5% of contingency for non-response rate, the minimum sample size required for this study was 292. Simple random sampling technique was employed to include participants into the study. From 292 participants approached, a total of sixteen patients were excluded from the study due to the unwillingness to give consent (8), incomplete medical record (6), and serious illness to complete the interview (2).

### Materials

A structured data collection tool that included a questionnaire and data abstraction checklist was used to extract all necessary information. The questionnaire included interview questions about socio-demographics, medication beliefs, knowledge of SCBs, and SCBs adherence while the data abstraction format included clinical and treatment related data.

Patients’ adherence to SCBs was assessed using Hypertension Self-Care Activity Level Effects (H–SCALE), which has been validated for use in several studies [27, 31, 42, 51]. H-SCALE is a self-reported questionnaire that contains six categories of SCBs including medication adherence, low salt intake, physical activities, smoking cessation, weight management, and alcohol abstinence. The categories of SCBs in H–SCALE are described as follows: Medication adherence contains three items with each score of 0–7 that summed up to give a total score ranging from 0–21. Low-salt intake contains nine items with each score of 0–7 which gives a total score ranging from 0–63. Physical activity was assessed by two items with each score of 0–7 that gives a total score ranging from 0–14. Likewise, weight management practice contains ten items based on a 5-point Likert scale ranging from 1 strongly disagree to 5 strongly agree, with a total score ranging from 10 to 50.

Patients’ beliefs about their medication were assessed using the belief about medicine questionnaire (BMQ) (**S1 Table**) [52]. BMQ has been validated and used in different chronic illness group studies including African population [32, 52–54]. BMQ includes two sections: BMQ-specific, which assesses the beliefs about the prescribed medications for personal use, and BMQ-general, which assesses the general beliefs about medications. We used BMQ-specific in this study. BMQ-specific is a self-reported questionnaire that encompasses two ‘five-item scales’ evaluating the patients’ belief about the necessity of their medicines for controlling their disease and their concerns about the potential adverse effects of taking medications. Participants indicate their degree of agreement with each statement on a five-point Likert scale, ranging from 1 strongly disagree to 5 strongly agree. Scores obtained for individual items within both scales are summed. Thus, the total scores for the necessity and concern scales range from 5 to 25. Higher scores indicate stronger beliefs. To determine the overall medication belief, a necessity–concerns differential is calculated as the difference between the necessity and the concern scales, with a possible range of -20 to 20.

### Study design and setting

An institutional based cross-sectional study design was conducted among ambulatory patients with HTN between January 2017 and March 2017 at the cardiac clinic of ACSH, Ethiopia. ACSH is the second largest public hospital in Ethiopia with a catchment population of about 10 million people.

### Procedure

We developed a questionnaire and data abstraction checklist to retrieve data from the patients and their medical record chart. The questionnaire was translated to local language (Tigrigna) and back translated to English for ensuring consistency. We employed four clinical nurses to collect the data for this study. Training and orientation were given for the data collectors. Pre-test was conducted on 5% of the sample before the commencement of the actual data collection and amendments were made on the data collection tool based on the findings. We recruited patients into the study during their appointment for medication refilling. Patients were interviewed to retrieve data regarding socio-demographics, medication belief, knowledge of SCBs, and adherence to SCBs. Respective medical and medication records were retrieved by reviewing the patient’s medical record chart using data abstraction checklist.

### Data analysis

Data were entered into an EPI data management (version 4.2.0) and analyzed using the Statistical Package for the Social Science (SPSS version 21.0). Descriptive analysis was computed using frequency and mean (standard deviation) for categorical and continuous variables, respectively. Multicollinearity was checked to test correlation among predictor variables using variance inflation factor and none was collinear. Univariable logistic regression analysis was performed to determine the association of each independent variable with SCBs. Furthermore, multivariable binary logistic regression model was done to identify predictors of SCBs. A p value of <0.05 was considered statistically significant in all analyses.

### Operational definitions

**Adherence to Self-care behaviors** is the extent of adherence of patients to the six categories of SCBs (medication adherence, physical activity, alcohol abstain, smoking cessation, low salt intake, and weight management), which are measured by H-SCALE (**S1 Table**). Categories of SCBs were defined and assessed based on H-SCALE [51] as follows:

#### Adherence to medication

Participants who took the recommended medication at the same time and took the recommended dose for 7 out of 7 days were said to be adherent to their medication.

#### Adherence to low-salt diet

Participants who took salt free diet while cooking and eating for at least 6 or more days out of 7 days were said to be adherent to low salt diet.

#### Adherence to physical activity

Participants who did physical activity at least 30 minutes for 8 or more days out of 14 days were said to be adherent to physical activity.

#### Alcohol abstinence

Participants who did not drink any alcohol in the last 7 days were said to be alcohol abstainers.

#### Smoking abstinence

Participants who did not smoke any pack of cigarette in the last 7 days were said to be smoking abstainers.

#### Adherence to weight management

Participants who scored 40 and above out of 50 were said to be adherent to weight management.

**Knowledge on self-care activities** is the extent of knowledge of the participants about the impact of SCBs on BP control and those who respond correct answer above the mean for knowledge related questions were considered as knowledgeable [41, 55].

**Medication belief** refers to the patient's attitude toward taking medication and description of wants, concerns, understandings, beliefs, and behaviors that are measured by BMQ [52]. Accordingly, participants were said to have **strong medication necessity belief** if the average sum of the 5-item medication necessity scale score (ranging from 5–25) is above the midpoint (>12.5); otherwise, they were said to have **poor medication necessity belief**. Similarly, participants were said to have **strong medication concern belief** about their medication adverse effect if the average sum of the 5-item medication concern scale score (ranges from 5–25) is above the midpoint (>12.5); otherwise, they were considered to have **poor medication concern belief**. The overall patients’ belief about their medication was determined by calculating the difference between the necessity and concern scale scores (ranging from -20 to 20). Hence, if the average sum of the 5–item medication necessity scale score exceeds the average sum of 5–item medication concern scale score, the patient is said to have **positive medication belief**; otherwise, the patient is said to have **negative medication belief.**

**Optimally controlled BP** was defined as an average systolic BP < 140 and diastolic BP < 90 mmHg for at least three consecutive follow-up appointments in patients younger than 60 years old or an average systolic BP< 150 and diastolic BP < 90mmHg if the age of patients was ≥ 60 years, otherwise, it was considered as **uncontrolled BP** [1].

### Ethics approval

This study was approved by the Ethics Review Committee of the School of Pharmacy, College of Health Sciences, Mekelle University. The aim and protocol of the study were fully elucidated to all study participants included in the study and written informed consent was obtained from each participant. The personal information was entirely confidential and protected. All methods were performed in accordance with the approved institutional guidelines.

## Results

### Socio-demographic characteristics

A total of 276 patients were included in the final analysis of the study. The mean (±standard deviation (SD) age of the participants was 52.54 ±15.6 years. More than half (52.5%) of the participants were females and about two-thirds (61.6%) were married. The majorities (80.1%) of the participants were urban dwellers. More than one-third (35.9%) of the participants were unable to write and read (Table 1).

**Table 1:** Socio-demographic characteristics of hypertensive patients in ACSH, 2017 (n = 276).

Characteristics	n (%)
Gender	
Female	145(52.5)
Male	131 (47.5)
Age in years	
18–35	29(10.5)
36–60	161 (58.3)
> 60	86(31.2)
Residence	
Rural	55 (19.9)
Urban	221 (80.1)
Educational level	
Unable to write and read	99 (35.9)
Primary education	75 (22.2)
Secondary education	39(14.1)
College and above	63 (22.8)
Marital status	
Married	170 (61.6)
Single	49(17.8)
Divorced	29(10.5)
Widowed	28(10.1)
Occupation	
Government employee	47 (17)
Non- government employee	33 (12)
Merchant	80(29)
Farmer	32(11.6)
House wife	84 (30.4)
Monthly income in Ethiopian Birr	
≤1500birr	145(52.5%)
>1500 birr	131(47.5%)
Khat chewers	27(9.8%)

### Medication beliefs

The majority (62%) of the participants had strong necessity belief towards their medication while 39.1% had strong concern belief towards their medication. Overall, only 36.6% had a positive belief about their medication (Table 2).

**Table 2:** Medication belief of hypertensive patients in ACSH, 2017 (n = 276).

Characteristics	n (%)
Medication necessity belief	
Strong necessity belief	171(62)
Poor necessity belief	105(38)
Medication concern belief	
Strong concern belief	108(39.1)
Poor concern belief	168(60.9)
Overall medication belief	
Positive belief	101(36.6)
Negative belief	175(63.4)

### Clinical and treatment related factors

Almost two-thirds (63.8%) of the participants had one or more comorbidities. One-fourth of the participants have lived with HTN for greater than five years and 36.2% had uncontrolled HTN. Nearly half (47.1%) of the participants were taking three and more drugs, which have been prescribed for the management of HTN or other comorbidities (Table 3).

**Table 3:** Clinical and treatment related characteristics of hypertensive patients in ACSH, 2017 (n = 276).

Characteristics	n (%)
Presence of comorbidity	
No	100(36.2)
Yes	176(63.8)
Duration of treatment	
<5years	207(75)
≥5years	69(25)
Number of drugs	
<3	146 (52.9)
≥3	130 (47.1)
BP checking per month	
<2 times	127(46%)
≥2 times	149(54%)
Hypertension control status	
Uncontrolled	100(36.2%)
Controlled	176 (63.8%)

### Knowledge about self-care behaviors

Most of the participants knew the negative effect of salt (94.9%), alcohol (81.5%), and smoking (87.7%) on the control of HTN while 54.7% knew the positive effect of physical exercise. Overall, 82.2% of the participants were knowledgeable about the impact of the SCBs on hypertension control (Table 4).

**Table 4:** Participants’ knowledge about the impact of SCBs on BP control among hypertensive patients in ACSH, 2017 (n = 276).

Characteristics	n (%)
Being overweight is risk to raise BP	208(75.4%),
Salt consumption raises BP	262(94.9%)
Physical exercise helps to reduce BP	151(54.7%)
Smoking cigarette has a negative effect on BP control	242(87.7%)
Drinking alcohol has a negative effect on BP control.	225(81.5%)
Medication is needed to treat hypertension	200(72.55)
**The overall knowledge about SCBs (n = 276)**	
Participants’ knowledge about SCBs	n (%)
Knowledgeable	227(82.2)
Not knowledgeable	49(17.8)

### Prevalence of adherence to self-care behaviors

**Fig 1:**
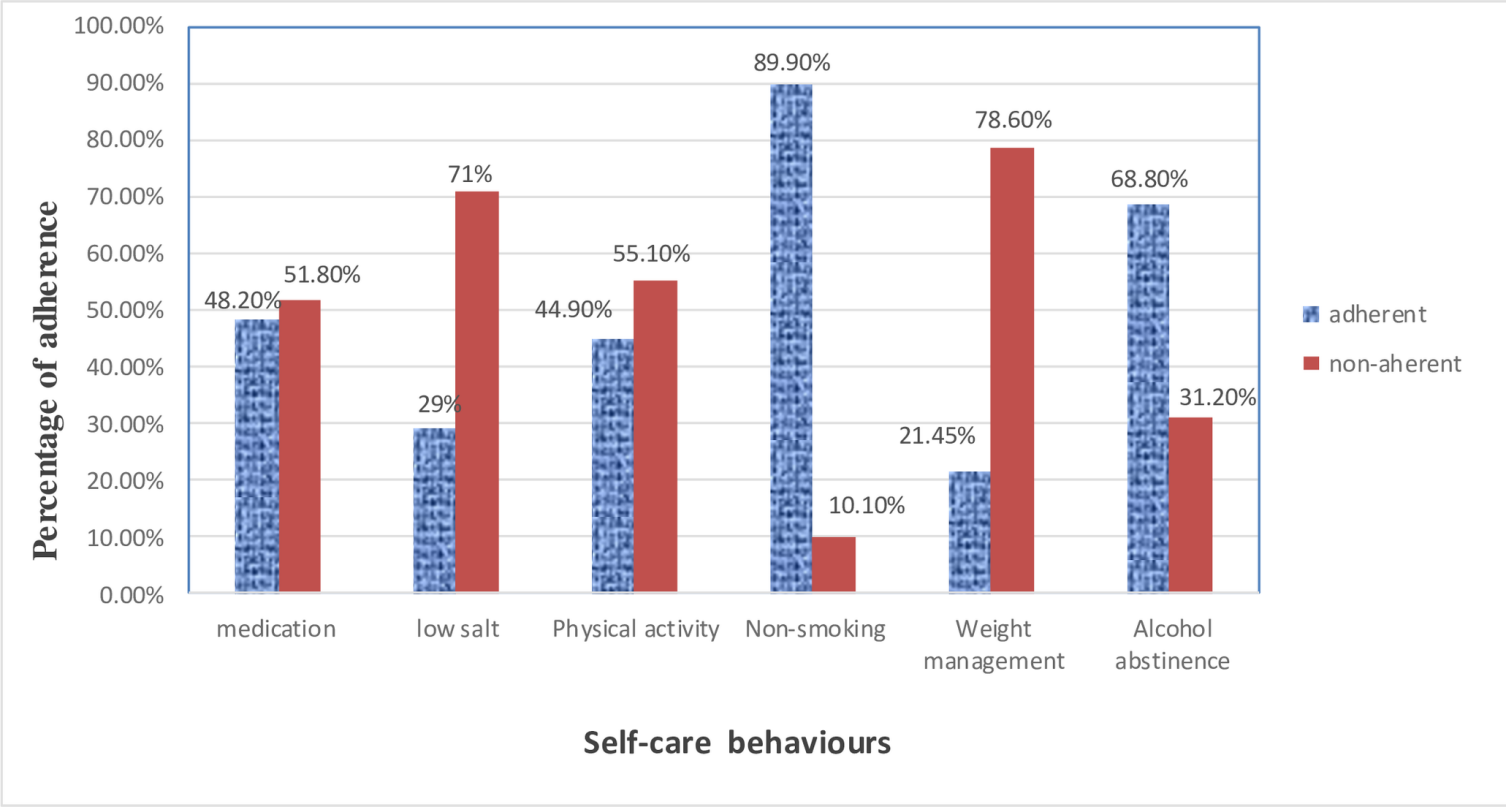
Prevalence of adherence to self-care behaviors among hypertensive patients in ACSH, 2017 (n = 276).

Our study revealed that the majority of the participants were nonsmokers (89.9%) and alcohol abstainers (68.8%). Less than half of the participants were adherent to the prescribed antihypertensive medications (48.2%) and the recommended level of physical activity (44.9%). Moreover, only 21.4% and 29% were adherent to weight management and low salt diet recommendations, respectively (Fig 1).

### Factors related to self-care behavior

The results of the multivariable logistic regression analysis (Table 5) indicated that females were less likely to adhere to weight management (Adjusted odds ratio (AOR): 0.46, 95% confidence interval (CI): 0.23–0.92) and physical activity (AOR: 0.22, 95% CI: 0.12–0.40) than males. However, females were more likely to adhere to alcohol abstinence (AOR: 1.97, 95% CI: 1.03–3.75) and non-smoking behavior (AOR: 6.33, 95% CI: 1.80–22.31) than males. Participants who were not knowledgeable on SCBs were less adherent to weight management (AOR: 0.13, 95%CI: 0.03–0.57) and alcohol abstinence (AOR: 0.07, 95% CI: 0.03–0.16) compared to those who were knowledgeable. Older participants (age > = 60 year) were less adherent to weight management (AOR: 0.19, 95% CI: 0.06–0.61) compared to the younger individuals (18–35 year). Khat chewers were less likely to adhere to non-smoking behavior (AOR: 0.08, 95% CI: 0.03–0.24) compared to the non-chewers. Rural residents were less adherent to their medication (AOR: 0.45, 95% CI: 0.21–0.97) than urban dwellers. Patients with comorbidity were also less adherent to their medication (AOR: 0.16, 95% CI: 0.08–0.31) than those without comorbidity. Moreover, patients with a negative medication belief were less likely to adhere to their medication (AOR: 0.25, 95% CI: 0.14–0.46) compared to those with a positive medication belief (Table 5).

**Table 5:** Factors associated with adherence to SCBs among hypertensive patients.

Characteristics	Medication adherence, AOR (95%CI)	Low-Salt diet adherence, AOR (95%CI)	Weight management, AOR (95%CI)	Physical activity, AOR (95%CI)	Alcohol abstinence, AOR (95%CI)	Non-Smoking, AOR (95%CI)
Gender, female	0.88(0.48–1.58)	0.87(0.48–1.60)	0.46(0.23–0.92)	0.22(0.12–0.40)	1.97(1.03–3.75)	6.33(1.80–22.31)
Age category						
18–35	1	1	1	1	1	1
36–59	1.13(0.39–3.28)	1.64(0.59–4.57)	0.43(0.17–1.09	0.59(0.24–1.50)	0.50(0.16–1.46)	0.25(0.03–1.90)
> = 60	0.88(0.48–1.58)	2.45(0.79–7.54)	0.19(0.06–0.60)	0.58(0.21–1.63)	0.91(0.26–3.14)	0.44(0.04–4.35)
Residence, rural	0.45(0.21–0.97)	1.16(0.55–2.46)	0.58(0.23–1.48)	1.04(0.51–2.11)	0.47(0.20–1.08)	1.54(0.31–7.63)
Education level						
Illiterate	0.53(0.20–1.43)	0.70(0.27–1.82)	0.50(0.16–1.60)	2.16(0.83–5.60)	1.74(0.61–5.00)	2.09(0.41–10.59)
Primary school	0.88(0.36–2.19)	0.50(0.21–1.20)	0.85(0.29–2.49)	1.30(0.55–3.11)	2.23(0.85–5.81)	1.58(0.38–6.57)
Secondary school	0.97(0.38–2.45)	0.90(0.37–2.191)	1.51(0.54–4.22)	1.02(0.41–2.53)	2.35(0.86–6.42)	2.27(0.54–9.53)
College and above	1	1	1	1	1	1
Monthly income in Ethiopian birr,						
<1500	1	1	1	1	1	1
> = 1500	0.88(0.41–1.85)	1.53(0.71–3.27)	0.39(0.16–0.93)	0.61(0.30–1.25)	0.55(0.23–1.31)	0.62(0.16–2.52)
Khat chewers	0.59(0.22–1.58)	1.39(0.56–3.43)	0.58(0.19–1.80)	1.80(0.71–4.55)	0.66(.24–1.76)	0.08(0.03–0.24)
Belief about medication						
Positive belief	1	1	1	1	1	1
Negative belief	0.25(0.14–0.46)	1.30(0.72–2.35)	1.43(0.71–2.90)	0.78(0.44–1.37)	1.28(0.66–2.45)	1.09(0.38–3.12)
Knowledge on SCBs						
Knowledgeable	1	1	1	1	1	1
Not knowledgeable	1.06(0.52–2.17)	1.37(0.70–2.72)	0.13(0.03–0.57)	0.83(0.41–1.65)	0.07(0.03–0.16)	1.13(0.33–3.81)
Duration of treatment in year						
< 5 years	1	1	1	1	1	1
> = 5 years	0.91(0.47–1.78)	1.19(0.63–2.23)	1.68(0.81–3.49)	1.03(0.56–1.90)	1.05(0.52–2.13)	0.58(0.20–1.74)
Comorbidity, yes	0.16(0.08–0.31)	1.39(0.73–2.67)	0.61(0.29–1.28)	0.70(0.38–1.30)	0.86(0.43–1.75)	0.60(0.18–1.99)
Number of medications						
<3	1	1	1	1	1	1
≥3	0.96(0.60–1.55	0.86(0.47–1.54)	1.38(0.68–2.81)	0.92(0.52–1.63)	1.02(0.53–1.96)	1.30(0.47–3.61)

Note: AOD–adjusted odds ratio, CI–confidence interval, SCB-self-care behavior

## Discussion

Engagement in SCBs is indispensable to control HTN and reduce complications and mortalities associated with HTN [1]. However, motivating patients to achieve high SCBs adherence is challenging, particularly in African population [56]. Assessment of patients’ adherence to SCBs and related factors is crucial to provide important information for clinicians working in the management of HTN. The findings of this study are helpful for healthcare professionals and policymakers in designing programs for future intervention. Therefore, our study determined the practice and predictors of SCBs among patients with HTN.

Our finding indicated that the majority of the patients were adherent to SCBs related to smoking and alcohol abstinence. However, the rate of adherence to SCBs like weight management, low salt intake, physical exercise, and medication was below 50%, which is comparable with the previous study conducted in Iran [31]. In contrast, a higher rate of adherence to the prescribed medication (61.3%), physical activity (52%), and low salt diet (81.1%) was reported from a study done in Beijing, China [42]. This variation could be due to the differences in the sociocultural factors and healthcare infrastructure. The potential explanation is that Ethiopian patients may have lower level of awareness about their disease and treatment than patients from Beijing[43]. Moreover, Ethiopian patients may have less exposure to western medicine compared to patients from Beijing, China.

In the present study, the rate of adherence to antihypertensive medications was 48.2% which is in line with the study conducted in the Democratic Republic of Congo (45.8%) [30]. In contrast, our finding was lower than the rate of adherence reported from the previous studies conducted in China (61.3%) and Ethiopia (64.6%) [42, 57]. On the other hand, our finding was higher compared to the rate of adherence reported from other studies done in India (24.1%) and South Africa (35%) [29, 44]. This could be attributed to the differences in the population demographics and disease distribution. For instance, all study participants were from rural population in the Indian and South African studies [29, 44] while 80% of the patients in the present study were from urban. Hence, the employment of rural residents in these studies might have contributed for the low rate of medication adherence. This could be due to the inadequate health literacy, lack of access to healthcare, and less exposure to western medicine in rural population [33, 58, 59].

Patients who had a negative medication belief were less likely to adhere to their medications compared to those with a positive medication belief. This finding was supported by other studies done in Ghana, Nigeria, and Pakistan [32, 49]. The potential source for negative medication belief in Africa could be due to the less awareness of patients on the benefit of western medicine and more trust in traditional medicine and faith healers [44, 46]. Patients with comorbidity were also less adherent to their medication compared to those without comorbidity which is comparable with the Bangladesh study [60]. This could be justified that patients with comorbidity are more likely to have multiple medications. Hence, they could be reluctant to take their medication appropriately [53]. Moreover, our finding indicated that rural residents had a lower rate of medication adherence than urban dwellers. This could be attributed to the lack of medication availability, low level of awareness about the disease and treatment, inability to afford medicines, mistrust in western medicine, and more trust on traditional and spiritual healers in the rural population [33, 58, 59, 61]. Therefore, we suggest provision of health education on the importance of medication adherence for HTN management in this population.

Scientific evidence revealed that eating low-salt diet is highly effective in reducing BP and CVD risk [13, 31, 62]. However, a low rate of adherence to the low-salt diet was reported in the current study. Similarly, a low rate of adherence was reported in other related studies [27, 40, 41]. In contrast to our study, a higher rate of adherence to the low salt diet was reported in China [42]. The reason for this variation could be due to the difference in feeding habit and ability of salt restriction. In Ethiopia, adding salt to make the food tastier is common practice [39]. Hence, future intervention should include educating and counseling patients about the importance of salt restriction for HTN control and on the ability of salt restriction.

Although different studies proved that losing weight by about 5–10 percent of one’s body weight has a positive impact on HTN treatment and CVD risk reduction [28, 63, 64], a low rate of adherence to weight management practice was reported in the present study. In line with our study, a low rate of adherence to weight management was reported in other similar studies [27, 40]. Lack of knowledge on SCBs was significantly associated with a low rate of adherence to weight management. This finding is also supported by another study done in Ghana [55]. Therefore, educational programs need to be designed to improve the knowledge of SCBs and weight management practice [65, 66]. Additionally, our finding indicated that female gender and old age (>60 years) were negatively associated with weight management. This could be explained that females and elders are less likely to take part in physical activities than their counterparts as evidenced by the world health organization (WHO) report [3].

The WHO has recommended at least 30 minutes of physical activity per day for five days per week to prevent and control high BP [13]. Despite the fact that regular aerobic exercise is helpful to reduce BP and prevent the complications of HTN [13, 17], less than half of the participants engaged in regular exercise in the present study. Similarly, a low rate of physical activity was reported from the previous studies done in Ethiopia and Jordan [40, 67]. Non-adherence to the recommended physical activity was more likely among females than males which is consistent with the Indian study [68].

In agreement with the previous studies done in Ethiopia and Nigeria [40, 69], the majority of the participants were alcohol abstainers in this study. In contrast, our finding is higher than a finding reported from Iran study [31]. This difference could be due to sociocultural difference as alcoholic drinks are banned in Iran while not in our setting [31]. Our finding revealed that women were more likely to abstain from alcohol compared to men. This finding is consistent with the study done among African Americans [27]. This could be attributed to the less sociocultural acceptance of alcohol consumption among females than males in our setting [70]. Furthermore, engagement in childcare and other family related activities may protect females from involving in alcohol consumption. Knowledge of SCBs was significantly associated with alcohol abstinence in our study. These findings suggest that future interventions should include education for patients on the risk of alcohol consumption and its negative impact on BP control.

In the present study, most of the patients were non-smokers which is consistent with previous studies done in Ethiopia and Nigeria [40, 69]. In contrary, a higher rate of smoking was reported from other studies done in African Americans and China [27, 42]. This might be due to the less sociocultural acceptance of smoking in Ethiopia [39]. We found a low rate of smoking in females than males which is in line with the findings of other similar studies [27, 42]. This could be attributed to the more pronounced sociocultural unacceptance of smoking among females than males in our setting [71]. Furthermore, Khat chewing was found to be an independent predictor of smoking behavior. This finding is comparable to the previous studies conducted in Ethiopia and Saudi Arabia [72, 73]. The possible reason for this could be explained that as both substances have a stimulant effect, they could be taken together to get the maximum excitement effect [72, 74].

## Limitations of the study

Finally, our study is not without limitations. The cross-sectional nature of our study may not provide adequate evidence of causality about non-adherence to SCBs and its risk factors. Due to self-report concerns, patients may understate socially undesirable activities like non-adherence to SCBs and negative medication beliefs. Moreover, the results of this study should be extrapolated to other countries with caution, as the study findings could be affected by the differences in the sociocultural factors, disease distribution, healthcare system, and healthcare team.

## Conclusion

The rate of adherence to hypertension SCBs particularly weight management, low salt intake, physical exercise, and medication intake was low in our study. Elders, females, khat chewers, rural residents, and patients with negative medication belief, comorbidity, and inadequate knowledge of SCBs were more likely to be non-adherent to SCBs compared to their counterparts. Hence, health care providers should pay more attention to patients at risk of having low SCB adherence. Patients should be educated and counseled about the importance of adherence to SCBs in the management of HTN. Healthcare providers and policymakers should design educational programs that are feasible to be implemented in populations with a low level of literacy and rural areas. Moreover, we recommend researchers to do further longitudinal studies with better study design to provide adequate information about cause and effect relationship between non-adherence to SCB and its risk factors.

## Supporting information

S1 Table Data collection instrument. (DOCX)

S2 Table Dataset. (RAR)
