# Supplementary material for: Practice and predictors of self-care behaviors among ambulatory patients with hypertension in Ethiopia
Source: PLoS One. 2019 Jun 26;14(6):e0218947. doi: 10.1371/journal.pone.0218947 (PMC6594646; doi:10.1371/journal.pone.0218947)
Supplement: S1 Table — (DOCX) [file pone.0218947.s001.docx]

**Data collection tool**

**Mekelle University**

**College of health science**

**School of pharmacy**

1. **Questionnaire English Version**

**Instruction: Tick (√) in provided number and fills the blank spaces by asking the patient.**

**Part I** - Participants’ Socio demographic Characteristics and awareness of hypertension related Variables

| S. no. | Questions | Alternative choices for Response | |
| --- | --- | --- | --- |
| 1 | Age | …… age | |
| 2 | Sex | 1. Male 2. Female | |
| 3 | Marital status | 1.Married  2.Single  3. Divorced  4.Widowed | |
| 4 | What is your religion? | 1. Orthodox 2. Muslim 3. catholic 4. protestant   _____________ | |
| 5 | What is the highest education level you completed? | 1. No formal education 2. Primary education (1-8 grade) 3. Secondary education (9-12 grade) 4. college and above | |
| 6 | What is your current occupation? | 1. government employee 2. NGO employee 3. Merchant 4. Farmer 5. House wife   Others (specify)_____ | |
| 7 | Ethnicity | 1. Tigray 2. Amhara 3. Affar 4. Oromo   Others specify ….. | |
| 8 | Residence | 1. Urban 2. Rural | |
| 9 | How much is your monthly income | ………………Birr(ETB) | |
| 10 | How long it had been since you were diagnosed with hypertension? | …………years/months | |
| 11 | How long it had been since you started taking anti-hypertensive drug? | ……… years/months | |
| 12 | Have you ever been told by a doctor or other health professional that you had hypertension before coming to start treatment? | 1. Yes 2. No | |
| 13 | How your current illness (HTN) for the first time was detected? | 1. During routine check-up 2. After complaint   Others, (specify)…………… | |
| 14 | Where do you monitor your blood pressure? | 1. Health center 2. Hospital 3. private clinic | |
| 15 | How many times per month do you check your blood pressure | ……............... | |
| 16 | What is average distance between your home and health institution you monitored? | ……………..(KM) |  |
| 17 | Currently, do you chew Khat? | 1. Yes 2. No |  |

**Part II. Knowledge on self-care activities (true /false)**

| NO. | Questions | Alternative Choices for Response | Skip to |
| --- | --- | --- | --- |
| 01 | Being overweight is risk to raise blood pressure. | 1. True 2. False 3. Don’t know |  |
| 02 | Salt consumption raises blood pressure. | 1. True 2. False 3. Don’t know |  |
| 03 | Physical exercise helps reduce blood pressure. | 1. True 2. False 3. Don’t know |  |
| 04 | Smoking cigarettes has a negative effect on persons with hypertension. | 1. True 2. False 3. Don’t know |  |
| 05 | Khat chewing has a negative effect on persons with hypertension. | 1. True 2. False 3. Don’t know |  |
| 06 | Drinking alcohol has a negative effect on persons with hypertension. | 1. True 2. False 3. Don’t know |  |
| 07 | A diet which contains fruits and vegetables is good for a person with hypertension. | 1. True 2. False 3. Don’t know |  |
| 08 | Which Complications of hypertension do you know**? (you can choose more than one)** | 1. Heart attack 2. stroke 3. renal failure 4. eye disease 5. stress 6. Others specify…… |  |
|  |  |  |  |

**Part III** - Hypertension—Self-care Activity Level Effects (H-SCALE) Items

| NO. | | Questions | Alternative Choices for Response | | Skip to | |
| --- | --- | --- | --- | --- | --- | --- |
| **Medication Usage**  How many of the past 7 days did you: from 0-7 days | | | | | | |
| 1 | Take your blood pressure pills? - | | |  | |  |
| 2 | Take your blood pressure pills at the same time every day? | | |  | |  |
| 3 | Take the recommended number of blood pressure pills? | | |  | |  |
| **Low-salt Diet**  How many of the past 7 days did you…from 0-7days | | | | | | |
| 4 | Follow a healthy eating plan as prescribed by physician? | | |  | |  |
| 5 | Eat potato chips, salted nuts, or salted popcorn? | | |  | |  |
| 6 | Eat smoked meats or smoked fish? | | |  | |  |
| 7 | Eat salted vegetables | | |  | |  |
| 8 | Eat ≥5 servings of fruits and vegetables? | | |  | |  |
| 9 | Eat store bought or packaged bakery goods? | | |  | |  |
| 10 | Salt your food at the table? | | |  | |  |
| 11 | Add salt to food when you’re cooking? | | |  | |  |
| 12 | Avoid eating fatty foods? | | |  | |  |
| **Physical Activity**  How many of the past 7 days did you…from 0-7days | | | | | | |
| 13 | Do at least 30 minutes total of physical activity? | | |  | |  |
| 14 | Do a specific exercise activity (such as swimming, walking, or biking) other than what you do around the house or as part of your work? | | |  | |  |
| **Smoking** | | | | | | |
| 15 | | Are you currently smoking? | | 1. yes 2. no | |  |
| 16 | | If yes, how many of the past 7 days did you smoke…from 0-7 days | |  | |  |

| **Weight management. 1=strongly disagree, 2=disagree, 3=neutral, 4=agree, 5=strongly agree** | | | | | | |
| --- | --- | --- | --- | --- | --- | --- |
|  |  | 1 | 2 | 3 | 4 | 5 |
| 17 | I am careful about what I eat. |  |  |  |  |  |
| 18 | I read food labels when I grocery shop. |  |  |  |  |  |
| 19 | I exercise in order to lose or maintain weight. |  |  |  |  |  |
| 320 | I have cut out drinking sugary sodas and sweet tea. |  |  |  |  |  |
| 21 | I eat smaller portions or eat fewer portions. |  |  |  |  |  |
| 22 | I have stopped buying or bringing unhealthy foods into my home. |  |  |  |  |  |
| 23 | I have cut out or limit some foods that I like but that are not good for me. |  |  |  |  |  |
| 24 | I eat at restaurants or fast food places less often. |  |  |  |  |  |
| 25 | I substitute healthier foods for things that I used to eat. |  |  |  |  |  |
| 26 | I have modified my recipes when I cook. |  |  |  |  |  |
| 27 | **Alcohol**  Did alcohol drink alcohol | | | 1. yes 2. no | |  |
| 28 | On average, how many days per week do you drink alcohol,(0-7) | | |  | |  |

**Part V. Assessment of Patient’s beliefs and concerns about their medicines**

- 1. Do you belief that your health, at present, depends on your medicines
- Strongly agree agree uncertain disagree strongly disagree
  1. Do you belief that your life would be impossible without your medicines

Strongly agree agree uncertain disagree strongly disagree

- 1. Do you belief that without your medicines you would become very ill

Strongly agree agree uncertain disagree strongly disagree

- 1. Do you belief that your health in the future will depend on your medicines

Strongly agree agree uncertain disagree strongly disagree

- 1. Do you belief that your medicines protect you from becoming worse

Strongly agree agree uncertain disagree strongly disagree

- 1. Do you belief that Having to take medicines worries you

Strongly agree agree uncertain disagree strongly disagree

- 1. Do you sometimes worry about the long-term effects of your medicines

Strongly agree agree uncertain disagree strongly disagree

- 1. Do you belief that your medicines are a mystery to you

Strongly agree agree uncertain disagree strongly disagree

- 1. Do you think that your medicines disrupt your life

Strongly agree agree uncertain disagree strongly disagree

- 1. Do you sometimes worry about becoming too dependent on your medicines

Strongly agree agree uncertain disagree strongly disagree

**Part VI: Clinical data review from registered follow up card of the patient**

| **1.** | Working diagnosis |  |
| --- | --- | --- |
| **2** | presence of comorbid cases? |  |
| **3** | Number of comorbidities |  |
| **4** | Prescribed medication regimen |  |
| **3.** | Type of drug ordered |  |
| **4.** | Number of drugs ordered/taken by the patient |  |
| **5.** | Blood pressure measured in the last three visits including data collection day | 1) 1st……………mmHg  2) 2nd…………..mmHg  3) 3rd……………mmHg |

1. **Questionnaire Tigrigna Version**

**ቃለ መሕተት ብቓንቓ ትግርኛ**

**ክፋል-1 ማሕበራዊ መነባብሮ ዝጥምቱ ሕቶታት**

| ተቑ | ሕቶ | መማረፂ |
| --- | --- | --- |
| 1 | ዕድመ | ዓመት |
| 2 | ፆታ | 1. ተባዕታይ 2. ኣንስታይ |
| 3 | ኩነታት ሓዳር | 1. ዝፈትሐ 2. ዝተመርዓወ 3. ዘይተመርዓወ 4. ዝፈትሐ |
| 4 | ሃይማኖት | 1. ኦርቶዶክስ 2. ፕሮቴስታንት 3. ሙስሊም 4. ካቶሊክ 5. ካልእ ይጠቐስ_____ |
| 5 | ደረጃ ትምህርቲ | 1. ዘይተምሃረ 2. ቀዳማይ ብርኪ 3. ካልኣይ ብርኪ 4. ልዕሊኡን |
| 6 | ስራሕ | 1. መንግስታዊ 2. ዘይመንግስታዊ 3. ነጋዳይ 4. ሓረስታይ 5. የቤት እመቤት 6. ካልእ ይጠቐስ____ |
| 7 | ብሄር | 1. ትግራይ 2. ኣምሓራ 3. ዓፋር 4. ኦሮሞ 5. ካልእ ይጠቐስ_____ |
| 8 | እትነብረሉ ቦታ | 1. ከተማ 2. ገጠር |
| 9 | ወርሓዊ እቶት | ……ብር |
| 10 | ደም ድፍኢት ካብ ዝርከበካ እዋን ጀሚሩ ክንደይ ገየርካ | …………ዓመት/ወርሒ |
| 11 | መድሓኒት መዐገሲ ደም ደፍኢተ ካብ እትጅምር ክንደይ እዋን ኮይኑካ | …………ዓመት/ወርሒ |
| 12 | ደም ድፍኢት ከም ዘለካ ብሓኪም ተነጊሩካ ዶ ይፈልጥ | 1. እወ 2. ኣይፋል |
| 13 | ሕማም ደም ድፍኢት ዝተነገረካሉ እዋን ትፈልጦ ዶ | 1. ንምርግጋፅ ኣብ ዝከድኩሉ እዋን 2.ድሕሪ ምሕማመይ 3. ካለእ ይገለፅ…………… |
| 14 | ደም ድፍኢትካ ኣበይ ክትትል ትገብር | 1. ኣብ ጥዕና ጣብያ 2. ኣብ ሆስፒታል 3. ኣብ ናይ ውልቀ ጥዕና ትካል |
| 15 | ኣብ ወርሒ ንክንደይ ግዘ በዝሒ ደም ድፍኢትካ ተረጋግፅ | ……............... |
| 16 | ካብ ገዛካ ናበ እትሕከመሉ ጥዕና ትካል ክንደይ ኪሎ ሜትር ይርሕቕ | …………….. ኪ.ሜ |
| 17 | 3ጫት ትቕሕም ዶ? | እወ ኣይፋልን |

**ክፋል-2 ኣፍልጦ ኣብ ዓርሰ ክንክን ዘድሀቡ ሕቶታት**

| ተቑ | ሕቶ | መማረፂ |
| --- | --- | --- |
| 01 | ክብደት ምውሳክ ንሕማም ድፍኢት ደም የቃልዕ | 1. ሓቂ 2. ሓሶት 3. ኣይፈልጥን |
| 02 | ብዙሕ ጠው ምጥቃም ድፍኢት ደም የጋድድ | 1. ሓቂ 2.ሓሶት 3. ኣይፈልጥን |
| 03 | ኣካላዊ መንቕስቃስ ምግባር ንድፍኢት ደም ይከላከለልካ | 1. ሓቂ 2.ሓሶት 3.ኣይፈልጥን |
| 04 | ሽጋራ ምትካክ ሕማም ድፍኢት ደም ንዘለዎም ሰባት ኣሉታዊ ፅዕንቶ ኣለዎ | 1. ሓቂ 2.ሓሶት 3. ኣይፈልጥን |
| 05 | ጫት ምሕያክ ሕማም ድፍኢት ደም ንዘለዎም ሰባት ኣሉታዊ ፅዕንቶ ኣለዎ | 1. ሓቂ 2. ሓሶት 3.ኣይፈለጥን |
| 06 | ኣልኮሆል ምስታይ ሕማም ድፍኢት ደም ንዘለዎም ሰባት ኣሉታዊ ፅዕንቶ ኣለዎ | 1.ሓቂ 2.ሓሶት 3.ኣይፈልጥን |
| 07 | ኣሕምልትን ፍራምረን ዘለዎ መግቢ ምጥቃም ሕማም ደም ድፍኢት ንዘለዎ ሰብ ኣዝዩ ንጥዕንኡ ሓጋዛይ እዩ. | 1. ሓቂ 2.ሓሶት 3.ኣይፈልጥን |
| 08 | ሕማም ድፍኢት ደም ናብ ዝለዓለ ጠርዚ ዝበፅሓሉ ኣብ እዋን ዝረኣዩ ሕማማት | 1. ሕማም ልቢ 2. ዝለዓለ ሕማም ኣእምሮ3. 3. ኩላሊት 4. ሕማም ዓይኒ 2. ጭንቀት 5. ካልእ…… |
|  |  |  |

**ክፋል-3 በዝሒ ደም መቓጻጸሪ ሕቶታት**

| ተቑ | | ሕቶ | መማረፂ | | | |
| --- | --- | --- | --- | --- | --- | --- |
|  |  |  | እወ | አይፋልን | | |
| **ኣጠቓቕማ መድሓኒት :** ኣብ ሸውዓተ መዓልቲ ክንደይ ማዓልቲ(0-7) | | | | | | |
| 1 | መድሓኒትካ ትወስድ ዶ | |  | | |  |
| 2 | መድሓኒትካ ማዓልታዊ ትወስድ ዶ | |  | | |  |
| 3 | ዝተኣዘዘልካ መድሓኒትካ ኩሉ ትወስድ ዶ | |  | | |  |
| ውሑድ ጨው ዘለዎ መግቢ ምምጋብ : ኣብ ሸውዓተ መዓልቲ ክንደይ (0-7) | | | | | |  |
| 4 | ሓኪም ብዝነገረካ ብትልሚ ዶ ትምገብ? | |  | | |  |
| 5 | ድንሽን ጨው ዘለዎን መግቢ ዶ ትምገብ? | |  | | |  |
| 6 | ዝተጠበሱ ምግቢታትን ዓሳን ዶ ትምገብ? | |  | | |  |
| 7 | ኣሕምልትን ፍራምረን ትምገብ ዶ? | |  | | |  |
| 8 | ካብ 5ን ልዕሊኡን ፍራምረ ትምገብ ዶ? | |  | | |  |
| 9 | ዝተዓሸጉ መግቢታት ትጥቀም ዶ? | |  | | |  |
| 10 | መግብኻ ጨው ገይርካ ዶ ትበልዕ? | |  | | |  |
| 11 | ፀብሒ ኣብ እትሰርሓሉ እዋን ጨው ትገብረሉ ዶ? | |  | | |  |
| 12 | ብዘይቲ ዝተጠበሱ መግቢታት ትበልዕ ዶ? | |  | | |  |
| 13 | ስብሒ ዝበዝሖም መግቢታት ካብ ኣመጋግባኻ ተወግድ ደ? | |  | | |  |
| **ኣካላዊ ምንቕስቓስ :** ኣብ ዝሓለፉ ሸውዓተ መዓልትታት(0-7) | | | | | | |
| 14 | ኣብ መዓልቲ ን30 ደቒቓ ኣካላዊ ምንቕስቓስ ትገብር ዶ? | |  | |  | |
| 15 | ካብ ገዛ ወፃኢ ፍሉይ ኣካላዊ ምንቕስቓስ ትገብር ዶ? | |  | |  | |
| **ሽጋራ ምትካኽ** | | | እወ | | አይፋል | |
| 16 | | ሀዚ ሽጋራ ተትክኽ ዶ? (0-7) |  | |  | |
| 17 | | ምልሶም እወ እንተኾይኑ ኣብ ዝሓለፈ ሰሙን ክንደይ ግዘ |  | |  | |

|  | **ክብደት ክትትል ምግባር** 1. ኣዝየ ኣይስማዕማዕን 2. ኣይስማዕማዕን 3. ኣይፈልጦን 4. ይስማዕማዕ 5. ኣዝየ ይስማዕማዕ | | | | |
| --- | --- | --- | --- | --- | --- |
| 18 | ብዛዕባ ዝበልዖ ይግደስ እየ | |  | |  |
| 19 | ኣብ ቤት መግቢ ኣብ ዝኸደሉ እዋን ዓይነታት መግቢ የንብብ እየ. | |  | |  |
| 20 | ኣካላዊ ምንቕስቓስ ዝገብር ክብደተይ ንምሕላው እዩ | |  | |  |
| 21 | ኣብ እዋን ምውሳድ መድሓኒት ክስተዩ ዘይብሎም ነገራት ጥሒሰ ሰትየ እየ. | |  | |  |
| 22 | ኣዝየ ይበልዕ እየ ወይ መግቢ ብዙሕ ኣይበልዕን | |  | |  |
| 23 | ጥዕና ዘይብሎም መግቢታት ምብላዕ ገዲፈዮ እየ | |  | |  |
| 24 | ዝፈትዎም ግን ድማ ንጥዕናይ ዘይሰማምዑኒ መግቢታት ምጥቃም ገዲፈዮ እየ | |  | |  |
| 25 | ኣብ እንዳ መግቢታት ከይድካ ምብላዕ ገዲፈዮ እየ | |  | |  |
| 26 | ዝምገቦም ዝነበርኩ መግቢታት ንጥዕናይ ሓገዝቲ ብዝኾኑ መግቢታት ተኪአዮም እየ | |  | |  |
| 27 | ዝወስዶ ሾርባ ኣመሓይሸ እየ | |  | |  |
| 28 | ኣልኮሆላዊ መስተ ዝጥምቱ ሕቶታት(0-7) |  | |  | |
| 29 | ኣብ ፍሉይ መዓልቲ ክንደይ ሰቲኻ? |  | |  | |
| 30 | ኣብ ዝሓለፈ ወርሒ ዝዓበየ በዝሒ መስተ ዝሰተኻዮ ክንደይ እዩ? |  | |  | |
| 31 | ጫት ትቕሕም ዶ? | እወ | | ኣይፋልን | |

**ክፋል 4 ኣብ ዓርሰ እምነትን አቀባብላን ዘድሀቡ ሕቶታት**

- 1. ጥዕናኻ ኣብ መድሓኒት እዩ ኢልካ ዶ ትኣምን

ኣዝየ ይስማዕማዕ ይስማዕማዕ ርግፀኛ ኣይኮንኩን ኣይስማማዕን ኣዝየ ኣይስማማዕን

- 1. ካብ መድሓኒት ወፃኢ ክነብር እየ ኢልካ ዶ ትኣምን

ኣዝየ ይስማዕማዕ ይስማዕማዕ ርግፀኛ ኣይኮንኩን ኣይስማማዕን ኣዝየ ኣይስማማዕን

- 1. ካብ መድሓኒት ወፃኢ ኣዝየ ክሓምም እየ ኢልካ ዶ ትኣምን

ኣዝየ ይስማዕማዕ ይስማዕማዕ ርግፀኛ ኣይኮንኩን ኣይስማማዕን ኣዝየ ኣይስማማዕን

- 1. ቀፃሊ ናብራኻ ኣብ መድሓኒት ዝተመስረተ እዩ ኢልካ ዶ ትኣምን

ኣዝየ ይስማዕማዕ ይስማዕማዕ ርግፀኛ ኣይኮንኩን ኣይስማማዕን ኣዝየ ኣይስማማዕን

- 1. መድሓኒት ሕማምካ ከይገደካ ዶ ይከላኸለልካ

ኣዝየ ይስማዕማዕ ይስማዕማዕ ርግፀኛ ኣይኮንኩን ኣይስማማዕን ኣዝየ ኣይስማማዕን

- 1. መድሓኒት ብምውሳድካ የተሓሳስበካ ዶ

ኣዝየ ይስማዕማዕ ይስማዕማዕ ርግፀኛ ኣይኮንኩን ኣይስማማዕን ኣዝየ ኣይስማማዕን

- 1. ንነዊሕ እዋን መድሓኒት ምውሳድካ ኣተሓሳሲቡካ ዶ ትፈልጥ

ኣዝየ ይስማዕማዕ ይስማዕማዕ ርግፀኛ ኣይኮንኩን ኣይስማማዕን ኣዝየ ኣይስማማዕን

- 1. እንታይ ዓይነት መድሓኒት ከምእትወስድ ንዓኻ ምሽጥር እዩ ዶ ትብል

ኣዝየ ይስማዕማዕ ይስማዕማዕ ርግፀኛ ኣይኮንኩን ኣይስማማዕን ኣዝየ ኣይስማማዕን

- 1. እትወስዶ መድሓኒት መነባብሮኻ የዛብዖ እዩ ዶ ትብል

ኣዝየ ይስማዕማዕ ይስማዕማዕ ርግፀኛ ኣይኮንኩን ኣይስማማዕን ኣዝየ ኣይስማማዕን

- 1. ኣብ መድሓኒተ ፅግዕተኛ ምኻንካ ኣተሓሳሲቡካ ዶ ይፈልጥ ዶ

ኣዝየ ይስማዕማዕ ይስማዕማዕ ርግፀኛ ኣይኮንኩን ኣይስማማዕን ኣዝየ ኣይስማማዕን
